# A Conversation with Raffaele Mezzenga

**DOI:** 10.1021/acscentsci.4c01773

**Published:** 2024-11-01

**Authors:** Carolyn Wilke

Raffaele Mezzenga has turned chicken feathers into fuel cell membranes, found myriad purposes for the milk protein whey, and used black bean proteins in aerogels for capturing carbon dioxide from air. Something of a hybrid between a materials scientist and a physicist, Mezzenga works at the Swiss Federal Institute of Technology (ETH), Zurich, finding new applications for food waste proteins.

Credit: Mohammad Peydayesh, ETH Zurich

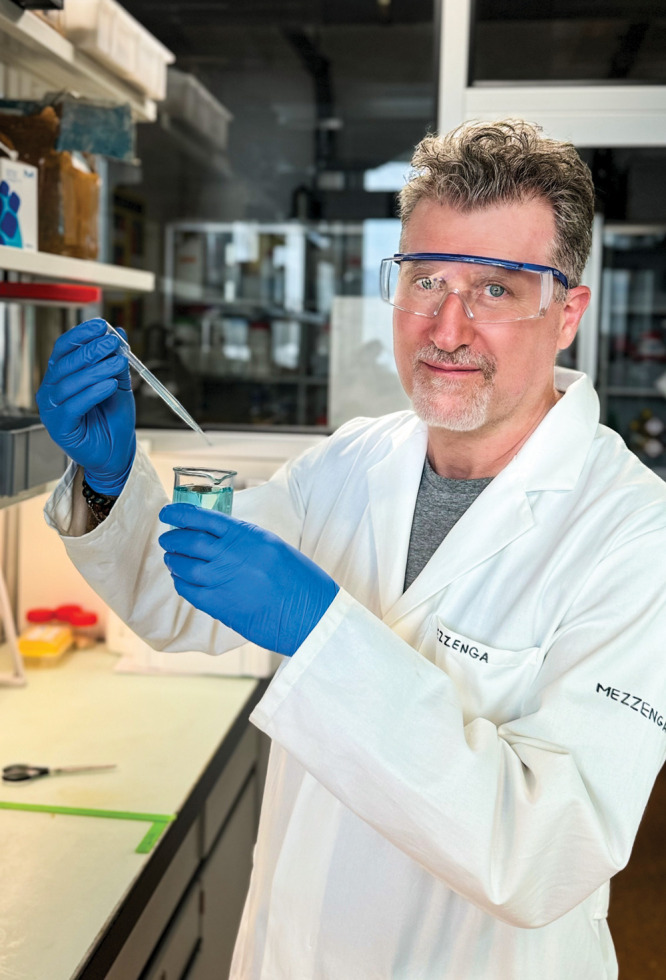

Mezzenga studied polymer physics, and his career quickly turned toward food. After a postdoctoral position, he moved to the Nestlé Research Center in Lausanne, Switzerland. There he started applying the know-how he’d gained from working with model synthetic polymers and colloids to polysaccharides, fats, and proteins—still often polymers and surfactants.

At Nestlé, Mezzenga started to manipulate proteins found in food waste into a form called amyloid fibrils to find useful ways of reusing them. Amyloids have a bad reputation because of their association with diseases such as Alzheimer’s, Parkinson’s, and type 2 diabetes. “There was a lot of reluctance, at least in the food industry, to use this splendid building block and put it back into food,” Mezzenga says. So Mezzenga, motivated by the problem of food waste, put his mind to finding other uses for amyloid fibrils.

Mezzenga has worked with proteins for decades, and his career may have now come full circle. Last year, he published research showing that mice can digest amyloid fibrils demonstrating the potential to use them in food. Following his time working at Nestlé, he’s transformed food proteins into materials for resource recovery, bioplastics, in vivo catalysis, water purification, and more. In the beginning, people thought he was absurd for his unorthodox applications for food waste proteins, Mezzenga says. “But I can say that, looking back at my career the last 20 years, I think it was a good bet from my side.”

Carolyn Wilke spoke with Mezzenga about the chemistry of amyloid fibrils and their potential applications. This interview was edited for length and clarity.

## Why repurpose proteins from food waste?

Proteins make up close to a third of food waste and are the most valuable component in food waste. You can extract polysaccharides and fats, but the chemical functionality that is provided by proteins is unchallenged. These are precision polymers, and they form primary, secondary, and tertiary structures. So they give a versatility in terms of chemistry that other components in food cannot give. If you extract this protein, you can process it, and most can be turned into amyloid fibrils, which will give you more functionality.

## What are amyloid fibrils?

Amyloid fibrils are a secondary structure of protein, a class of protein aggregate. They were discovered in the context of neurodegenerative diseases, such as Alzheimer’s and Parkinson’s. They used to be the bad guys. Now they are actually building blocks for complex materials and technologies. To get amyloid fibrils, you unfold the protein and break it down into pieces. Fragments of the protein precursor self-assemble to form zigzags called β-strands. β-strands stack on top of each other and form β-sheets. And then the β-sheets, they stack one on top of each other, and they form the fiber. In the amyloid fibril, the hallmark is that β-strands are orthogonal to the fiber axis.

## What are the properties of amyloid fibrils that make them useful?

The chemical properties of amyloid fibrils are the same as native protein. The most striking difference is in the physical properties. The nanometer-scale polypeptides are very flexible, but they form micron-sized fibers that are extremely rigid. The Young’s modulus, or stiffness, is on the order of gigapascals—similar to that of most commodity plastics. Most [synthetic] polymers only have a few monomers. With these fibers, think of having a material that has mechanical properties comparable to plastics but with versatile chemistry offered by 20 different amino acids.

When you turn a protein from a folded protein into nanofibers, the amino acids become more available to do some chemistry. For instance, these amino acids may act as a ligand to interact with metal ions. If you make a membrane out of this fiber and pass water through it, the metal will be transferred to the protein membrane. Whatever the metal is—say lead, gold, palladium, or mercury—there will be an amino acid that has strong binding properties. If you are comparing this to water purification technology, it is like having 20 ion exchange resins working together. It removes large objects like parasites or bacteria, and we can modify the fibers with iron to adsorb viruses. Gold ions are adsorbed splendidly by the same amyloid gels. You can then just convert these ions into gold nuggets by using a thermal reduction. So that is a way to handle recovering gold from e-waste.

**Figure:**
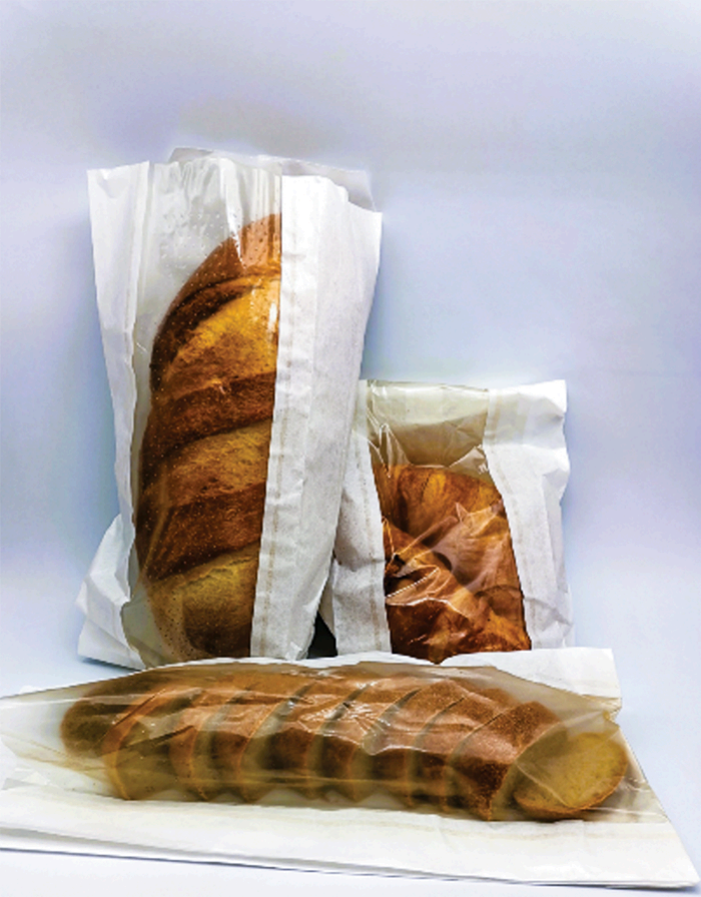
Raffaele Mezzenga's team has transformed soy waste, such as the liquid left over from producing tofu, into transparent film that can be used for food packaging. Credit: *Biomacromolecules*

## What are some other ways that you want to apply waste proteins?

We’re talking about in vivo applications. These fibers can be used to adsorb and deliver bioavailable iron. Also, we have coordinated an atom of iron on the protein fibril that keeps the iron [in a particular coordination state] that is similar to horseradish peroxidase. So it is capable of performing oxidase reactions. This is now a beautiful technology for alcohol detoxification directly in the gut. This amyloid fibril is able to catalyze the oxidation of ethanol into acetic acid in mice. We are working hard on that to make the formulation translatable to humans.

What else is left on the checklist that humanity should tackle as soon as possible? Renewable energy. We use keratin amyloid fibrils that we extract from chicken feathers to make fuel cell membranes. The performance is good. It is certainly not as good as a perfluorinated polymer like Nafion. But these membranes are nontoxic, biodegradable, recovered from food waste, and they will cost 5–10 times less than a fuel cell membrane. And this is a classic example of protein from food waste that cannot be reintroduced in the food value chain, because keratin has very poor nutritional value.

## What applications have you commercialized?

We have commercialized two technologies. One is a company called BluAct [Technologies] using amyloid fibrils for water purification. There is another company called Goold using amyloid fibrils to grow gold in a specific plane to make single crystals. I am a cofounder of both. Now we [Mezzenga and colleagues] are working very hard to bring to the market two other technologies: the recovery of gold from e-waste and alcohol detoxification.

## What are some of the barriers to commercialization?

They are specific to the application. For gold from e-waste, the value of gold is so high that it is very straightforward to implement the technology. But in the case of bioplastics, the major hurdle is that the cost of commodity plastic is so cheap that it is hard to change people’s mindset.

From a technical point of view, one of the biggest [challenges] is to make a water bottle you can drink from [that will] biodegrade when you throw it away. It has to be hydrophobic because otherwise you cannot use it to store water, and hydrophilic because it has to dissolve and degrade in a wet environment. There are [commercial] biodegradable polymers that can do some of this, but the price is still high compared with our bioplastics.

## What do you wish other chemists knew about these materials?

I invite people to have fun and think out of the box to imagine other possible applications. These proteins are available at basically no or very little cost. We produce one gigaton of food waste, of which about one third is protein, every year. The scale is comparable to the amount of plastics we make. By the time the protein has been wasted, the carbon footprint is already there. So we better reuse that to do something that we will otherwise use neat, pristine materials for.

## Carolyn Wilke is a freelance contributor to

Chemical & Engineering News, *the independent news outlet of the American Chemical Society*.

